# Assessment of Canopy Conductance Responses to Vapor Pressure Deficit in Eight Hazelnut Orchards Across Continents

**DOI:** 10.3389/fpls.2021.767916

**Published:** 2021-12-08

**Authors:** Gaia Pasqualotto, Vinicio Carraro, Eloy Suarez Huerta, Tommaso Anfodillo

**Affiliations:** ^1^Forest Ecology Research Unit, Department of Territorio e Sistemi Agro-Forestali, Università degli Studi di Padova, Legnaro, Italy; ^2^Hazelnut Company Division, Ferrero Trading Luxembourg, Findel, Luxembourg

**Keywords:** stomatal sensitivity, sap flow, orchard management, global warming, *Corylus avellana* (L.)

## Abstract

A remarkable increase in vapor pressure deficit (VPD) has been recorded in the last decades in relation to global warming. Higher VPD generally leads to stomatal closure and limitations to leaf carbon uptake. Assessing tree conductance responses to VPD is a key step for modeling plant performances and productivity under future environmental conditions, especially when trees are cultivated well outside their native range as for hazelnut (*Corylus* spp.). Our main aim is to assess the stand-level surface canopy conductance (*G*_*surf*_) responses to VPD in hazelnut across different continents to provide a proxy for potential productivity. Tree sap flow (*Fd*) was measured by Thermal dissipation probes (TDP) probes (six per sites) in eight hazelnut orchards in France, Italy, Georgia, Australia, and Chile during three growing seasons since 2016, together with the main meteorological parameters. We extracted diurnal *Fd* to estimate the canopy conductance *G*_*surf.*_. In all the sites, the maximum *G*_*surf*_ occurred at low values of VPD (on average 0.57 kPa) showing that hazelnut promptly avoids leaf dehydration and that maximum leaf gas exchange is limited at relatively low VPD (i.e., often less than 1 kPa). The sensitivity of the conductance *vs*. VPD (i.e., -d*G*/dlnVPD) resulted much lower (average *m* = −0.36) compared to other tree species, with little differences among sites. We identified a range of suboptimal VPD conditions for *G*_*surf*_ maximization (*G*_*surf*_ > 80% compared to maximum) in each site, named “VPD_80_,” which multiplied by the mean *G*_*surf*_ might be used as a proxy for assessing the maximum gas exchange of the orchard with a specific management and site. Potential gas exchange appeared relatively constant in most of the sites except in France (much higher) and in the driest Australian site (much lower). This study assessed the sensitivity of hazelnut to VPD and proposed a simple proxy for predicting the potential gas exchange in different areas. Our results can be used for defining suitability maps based on average VPD conditions, thus facilitating correct identification of the potentially most productive sites.

## Introduction

The role of the leaf to air vapor pressure deficit (VPD) is increasingly recognized as a leading limiting factor to determine plant gas exchange (Novick et al., 2016; Grossiord et al., 2020), while the influence of climate change on VPD rise becomes more and more evident. Anthropogenic-related global warming has increased unceasingly by 0.2°C every decade for more than 30 years (IPCC, 2018). A sharp increase of VPD was observed in the last decades (Yuan et al., 2019) and climatic projections show a future scenario where VPD increase is a diffuse phenomenon in many areas of the globe (Ficklin and Novick, 2017; Barkhordarian et al., 2019).

Higher VPD is in general detrimental for plant productivity because of the negative exponential response of stomatal conductance *g*_*s*_ to increasing VPD (Schulze et al., 1972; Jones, 1992; Oren et al., 1999). This response limits plant dehydration, but, at the same time, reduces carbon assimilation once stomata are partially closed. This explains why VPD was identified as a climatic factor related to tree mortality (McDowell et al., 2013) and limits crop productivity (Eamus et al., 2013). Prolonged periods of high VPD have been related to reduced growth (Sanginés de Cárcer et al., 2018) or large-scale forest die-off (Breshears et al., 2013; Will et al., 2013). Similarly, major crops such as maize, wheat, soybean, and fruit trees were reported to have decreased yields in response to high VPD (Challinor and Wheeler, 2008; Lobell et al., 2011; Zhao et al., 2017; Hsiao et al., 2019). Because plants are at the base of human life, the impact of changing climate and VPD rise on food provided by plants are a main issue in the upcoming future.

The response of plants to VPD variation has been extensively studied both at the leaf and at stand level. The latter is more suitable for assessing the effect of different management systems and structures in a specific site/orchard. At stand level, the surface canopy conductance *G*_*surf*_ (mol m^–2^s^–1^) refers to the capacity of gas exchange of the whole canopy expressed per unit of ground area, which includes a wide variety of heterogeneous leaf conditions (Medrano et al., 2015) and the community structure in terms of leaf area, roughness, soil properties, and species (Grossiord et al., 2020). In stands where the decoupling coefficient Ω is low (Jarvis and McNaughton, 1986), stomata experience a VPD close to the free atmosphere and the total canopy conductance is assumed to be driven largely by stomata aperture (Köstner et al., 1992; Hogg and Hurdle, 1997; Chapin et al., 2002). Thus, canopy conductance *G*_*surf*_ can be estimated from the rate of canopy transpiration (*Ec*) and VPD simply as: *G*_*surf*_ = k × *Ec*/VPD (Köstner et al., 1992; Arneth et al., 1996) where k is a temperature-dependent parameter (see below). *G*_*surf*_ includes two components: the stomatal component (average of different canopy leaves) and the aerodynamic component, which is a function of the orchard structure (i.e., density, height, roughness). Thus, when it is considered on a relatively long-term scale (weeks, months), *G*_*surf*_ can be a proxy for carbon uptake of the community, which, in turn, should also determine the carbon pool for reproductive allocation and seed production. In its turn, maximum value of *G*_*surf*_ (i.e., *G*_*max*_) indicates the potential gas exchange of the whole canopy.

The potential conductance is reached under non-limiting conditions of soil water availability, light, and optimal temperature. In forests, these conditions are rarely met and *G*_*max*_ is generally estimated. Thus, Oren et al. (1999) proposed to use the *G*_*surf*_ reached at 1 kPa (G_*surf*@1k*Pa*_) as reference maximum conductance. G_*surf*@1k*Pa*_ is highly variable across ecosystems because it depends both on the stand characteristics and species. In natural forest formations, it ranges from 0.2 to 0.7 mol m^–2^s^–1^ from savannah to deciduous forests (Grossiord et al., 2020). Tang et al. (2006) reported *G_*surf*@1k*Pa*_* 0.024 (mol m^–2^s^–1^) for some broad leaves of the genera *Betula, Acer*, and *Ostrya* and Fernández et al. (2009) from 0.28 to 0.44 in *Nothofagus antarctica* and *Diostea juncea*. On conifers, Köstner et al. (1996) and Köstner et al. (1996) reported *G_*surf*@1k*Pa*_* = 0.16–0.4 (mol m^–2^s^–1^) in *Pinus sylvestris*.

At the same time, some studies showed that many species, including hazelnut, present *G*_*max*_ occurring before 1 kPa (Tang et al., 2006; Herbst et al., 2008). Some other species reach *G*_*max*_ even above 1 kPa such as for example *Pseudotsuga menziesii*, where the *G*_*max*_ = 0.64 (mol m^–2^s^–1^) is reached at about 1.8 kPa (Fernández et al., 2009). Also some studies on leaves of fruit trees as olive (Rodriguez-Dominguez et al., 2019), walnut (Rosati et al., 2006), and different apple cultivars (Massonnet et al., 2007) show that *G*_*max*_ is reached between 1.5 and 2 kPa, probably related to the incidence of the fruit load in the whole tree physiology. Indeed, a correct approach to define the maximum potential gas exchange would be to consider the actual *G*_*max*_, which may occur at different VPD according to the species or stand management.

Still, the reference *G_*surf*@1k*Pa*_* is often used to determine the sensitivity of a species. The sensitivity of stomatal response (even at canopy level) to VPD refers to the relative reduction in *G* with increasing VPD (i.e., -d*G*/dlnVPD). It is commonly believed that high *G_*surf*@1k*Pa*_* predicts high sensitivity (Oren et al., 1999). The sensitivity is measured as the slope (*m*) of the function *G*_*surf*_ = *G_*surf*@1k*Pa*_* – *m* lnVPD. *G_*surf*@1k*Pa*_* and *m* seem highly correlated (average *R*^2^ = 0.75) with a slope of approximately 0.6 across species. In other words, the higher the *G_*surf*@1k*Pa*_*, the faster the stomata close with VPD increase, compared to species or individuals that present a lower *G_*surf*@1k*Pa*_*. However, it is likely that the sensitivity changes whether we consider *G_*surf*@1k*Pa*_* or *G*_*max*_.

In this study, we explored the relationship between VPD and whole tree conductance *G*_*surf*_ as a contribution to understanding the limitations of potential productivity in eight commercial hazelnut orchards distributed in different countries. These sites have been considered as eight representative orchard conditions in a range of bearable climates for hazelnut growth, going from mild temperate to dry warm. Increasing VPD connected to global warming (IPCC, 2018; Barkhordarian et al., 2019) will certainly threaten these areas. Thus, we aimed at: (I) identifying the maximum canopy conductance and its sensitivity to VPD at each site and (II) providing a procedure to quantify the maximum potential gas exchange of a site by knowing the frequency of occurrence of VPD values.

The assessment of critical thresholds of VPD that guarantee optimal *G*_*surf*_ will become of great importance in defining suitability maps to locate new plantations and predict the responses of current crops to future climate change.

## Materials and Methods

### Study Areas, Orchard Characteristics, and Experimental Setting

This study occurred in eight different commercial orchards of European hazelnut (*Corylus avellana*, L.) distributed in both the hemispheres. In the northern hemisphere: in France (Cancon, 44°18′N, 0°34′E, named “F1”), Italy (Baldissero d’Alba, 44°45′N, 7°55′E, named “I1”), and Georgia (Gejeti, 42°19′N, 42°12′E, named “G2”); in the southern hemisphere: in Chile (Camarico, 35°18′S, 71°21′W, named “C1”; San Sebastian, 35°17′S, 71°32′W, named “C2”), and Australia (Glendale farm, 34°48′S, 146°40′E, named “A0”; Narrandera, 34°48′S, 146°40′E, named “A1”; Orange, 33°19′S, 149°5′E, named “A2”). The experiment included three main commercial cultivars: Tonda Trilobata (TT), Tonda di Giffoni (TG), and Ennies. Table 1 presents the biometrics and orchard features for all the study areas. The differences of orchard characteristics between sites are representative of diverse types of orchard management systems of the species. The total tree basal area refers to the sum of basal area of all the sprouts belonging to each single individual tree, which is normally grown as multistem. The leaf area index (LAI) derives from estimation of the tree leaf area (m^2^), which, in turn, is derived by a species-specific allometric equation, namely *leaf area = 0.16 × D^2.22^* (*R*^2^ = 0.93), where *D* is the diameter (in cm) at the base of each stem. The relationship was established by using both the cultivars TT by Pisetta (2012) and TG (data not published). These orchards all entered the productive stage. Because hazelnut has a strong alternance in fruit bearing between years (almost two times of magnitude between years) and the fruit load (kg/tree) in Table 1 is the maximum value recorded in the three growing seasons in the parcel of the site where the trial was located. Thus, the data reported to the tree level shall be considered as an estimation of the maximum potential.

**TABLE 1:** Tree biometrics in the study areas: tree spacing, estimated tree height, sprout number per individual tree, total basal area (SD in brackets), estimated leaf area index (LAI), fruit load (maximum value recorded in the period), cultivar (TG, Tonda di Giffoni; TT, Tonda Trilobata; E, Ennies), and training system.

Site	Tree spacing	Tree height		Sprout n.		Tree basal area		LAI	Fruit load	Cultivar	Training system
	(m)	(m)		(n.)		(dm^2^)			Kg/tree		
A0	4x5	3.2	(0.25)	9.8	(6.11)	0.69	(0.19)	0.99	2	E	Single stem
A1	4x5	2.1	(0.15)	7.5	(2.07)	0.53	(0.10)	0.70	1	TG	Multi stem
A2	4x5	3.2	(0.03)	4.3	(0.51)	0.94	(0.09)	1.39	n.a.	TG	Multi stem
C1	6x5	2.8	(0.27)	7	(1.10)	1.87	(0.16)	1.90	5.9	TG	Multi stem
C2	4x6	4.5	(0.12)	5.8	(1.72)	2.23	(0.24)	3.05	7.4	TT	Multi stem
F1	3x5	5.2	(0.27)	3.11	(0.88)	2.65	(0.37)	6.10	7.9	TG	Single stem
G2	3x5	3.5	(0.80)	11	(3.35)	1.43	(0.36)	2.06	4.5	TG	Multi stem
I1	5x5	3.8	(0.12)	6.5	(1.64)	2.44	(0.54)	3.12	5.5	TT	Multi stem

*Non-available data = na.*

These orchards all entered the productive stage. Trees were in their productive stage in all the orchards (between 5 and 10 years old). Orchards are subjected to standard agronomic practices to support nut production (Roversi, 2014). Irrigation starts just before the nut cluster formation with about 3 mm of water per day distributed by a drip irrigation system. This study covered three growing seasons from 2016 to 2019. Sap flow was monitored by mean of thermal dissipation probes Granier type, 20 mm long (self-made in the Department of Territorio e Sistemi Agro-Forestali lab). Details on the construction and installation of Thermal dissipation probes (here after TDPs) are given in Pasqualotto et al. (2019). TDPs were installed in six trees per site in the phenological phase V03–V04 (bud brake and leaf emergence). Hazelnut phenology was collected at the orchard level according to a protocol proposed by Ferrero Agri-Farms, built on the (Biologische Bundesanstalt, Bundessortenamt and Chemical Industry, i.e. the German scale used to identify the phenological development stages of a plant) and on Romano et al. (1998). TDPs were set north-east facing on one branch per tree at about 50 cm above the ground in order to avoid thermal disturbance or humidity from the soil and possible damages to the sensors from the mechanical removal of sprouts. Further, TDPs were thermally insulated with aluminum heat insulation foil and Styrofoam. Two soil water content probes measuring the volumetric water content (TDR probe, Mod. CS650, Campbell Scientific Incorporation, Logan, USA) were installed between monitored trees at 30 and 60 cm depth. A data logger (CR1000, Campbell Scientific Incorporation, Logan, United States) recorded all the data every 15 min over the entire growing season, i.e., up to V08, leaves senescence, and shedding. A solar panel and a battery provided constant power supply to the stations. Air temperature (°C) and relative humidity (RH%) were recorded right on top of tree crowns in the orchard. Sap flow probes were replaced at the beginning of each growing season to prevent signal decaying.

### Data Elaboration

The sap flow density *Fd* (dm^3^ dm^–2^ h^–1^) was calculated by using the classic equation form Granier (1985):


(1) $$Fd=a\cdot k^{b}$$

*k* = (ΔT0–ΔT)/ΔT, where ΔT is the temperature difference between the two probes (the heated vs. the reference one), ΔT0 is the maximum temperature difference (i.e., the condition corresponding to zero flow). For this study, we used specific parameters calibrated for hazelnut *b* = 1.45 and the constant *a* = 13.86 (Pasqualotto et al., 2019). Canopy transpiration (*E*_*C*_) (g m^–2^ s^–1^) was calculated from mean *Fd* by multiplying it with the total sapwood area (*A*_*S*_) over the orchard ground surface (*A*_*O*_) (Oren et al., 1998):


(2) $$E_{C}=Fd\cdot \frac{A_{S}}{Ao}$$

The total water vapor transfer capacity or conductance at stand/orchard level (*G*_*surf*_) in mm s^–1^ was estimated from the canopy transpiration *E*_*C*_ and air VPD (Tang et al., 2006):


(3) $$G_{surf}=k\cdot E_{C}/VPD$$

Where k = 115.8 + 0.4226 *T* (m^3^ kPa °C kg^–1^), *T* is the temperature (°C), and VPD = *es* – *ea* = *es* – (RH × *es*/100), where *ea* is a function of the relative humidity expressed in percent (Monteith and Unsworth, 2013). This was converted to mol m^–2^ s^–1^ according to Jones (1999) to allow for comparisons with recent reports. We also calculate the same values per unit of leaf area as EL = E_*c*_/LAI and *G*_*surf*_ per unit of leaf area. The data set were subset to obtain diurnal data from 6 a.m. to 6 p.m. for the growing season: May to August for the northern hemisphere and November to February for the southern hemisphere. This choice means to extract the transpiration values related to positive carbon dioxide (CO_2_) assimilation condition. To compare the slopes of the response curve between *G*_*surf*_ and VPD, we calculated the relative value of canopy conductance *G*_*rel*_:


(4) $$G_{rel}=G_{surf}/G_{max}$$

where *G*_*max*_ is the maximum value of *G*_*surf*_ that occurs in the interval VPD_80_. For the sake of better understanding of the site dynamic, data have been aggregated per site by averaging the response of G_*rel*_ to VPD of multiple growing seasons. An ANOVA was applied to the dataset to check for differences of stomata sensitivity between sites. *G*_*rel*_ is averaged into VPD classes of 0.1 kPa to simplify the response analysis in a continuous pattern (Hogg and Hurdle, 1997). The response of *G*_*rel*_ to VPD site by site aims at defining which are the best conditions for gas exchange around the optimum (*G*_*max*_) in each study area. To meet this target, values of *G*_*rel*_ > 80% were selected and the corresponding VPD values were defined in a specific range of VPD at site level (VPD_80_). Every time trees experience VPD belonging to the VPD_80_ range and we can assume that canopy gas exchange was relatively high.

**FIGURE 1:**
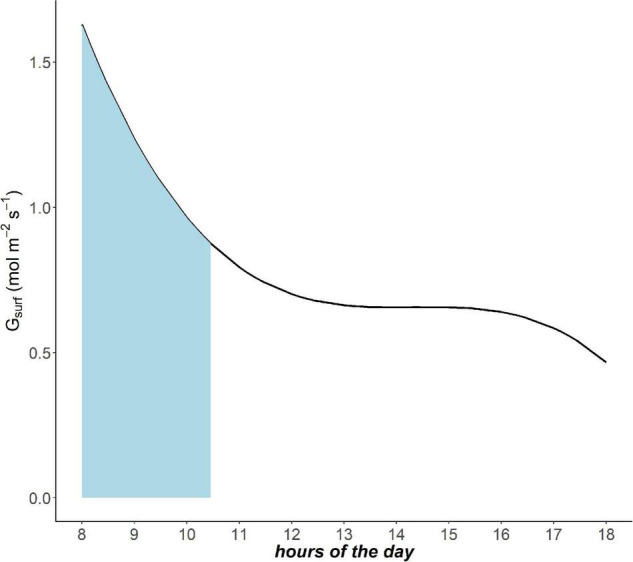
Example of daily pattern of canopy conductance *G*_*surf*_ (mol m^– 2^s^– 1^) in site I1 day of the year 175 (i.e., Italy 24th June). The light blue area underlies the time interval in which vapor pressure deficit (VPD)_80_ occurs.


Trees experience conditions of VPD_80_ at different times during the day throughout the year. The frequency of VPD_80_ over the growing season in a specific site is critical to determine the total achievable gas exchange capacity (GEC). In order to include the temporal factor, we calculated the integral of the daily *G*_*surf*_ curve only in the intervals of VPD_80_ occurrence (e.g., in Figure 1 for day of the year 175 in site I1—Italy). The sum of these integrals at site level for the entire growing season gives the GEC:

$$GEC=n.VPD_{80}\cdot G_{surf80}$$

Where n.VPD_80_ is the number of occurrences (expressed in seconds) of VPD_80_ per site during each growing season (mean occurrences per growing season) and *G*_*surf80*_ is the mean value of *G*_*surf*_ recorded in the VPD_80_ at each site. We obtained an estimation of the total GEC of a growing season at site level according to the orchard characteristics and to the average climatic conditions (Mmol m^–2^).

## Results

### Climatic Conditions

The mean daily air VPD condition during the growing season ranged widely from 1.01 kPa day^–1^ in site C1 and G2 to 1.93 and 2.66 kPa day^–1^ in the Australian sites A0 and A1, respectively (Table 2) (Kruskal–Wallis nonparametric test, *p* < 0.001). Site A1 also resulted the warmest with peaks of 46°C, while C1 the coolest with minimum reaching −5°C during the growing season. The global radiation was higher in southern hemisphere sites, especially in Chile (sites C1 and C2) (about 30 MJ m^–2^ day^–1^), while in northern hemisphere sites, the global radiation was generally lower (about 20 MJ m^–2^ day^–1^). The volumetric water content (VWC) of the soil ranged between 0.09 and 0.45 m^3^ m^–3^ and it is linearly correlated (*R*^2^ = 0.75) with the mean VPD at site level, being highly dependent on the site temperature. The remaining difference can be explained by the soil texture, which is, for example, high in sandy soil in site A1.

**TABLE 2:** Main climatic parameters at each site during the growing season (May to August or November to February).

Site	Mean VPD	Air temperature			Global radiation	VWC
	(kPa)	min (°C)	mean (°C)	max (°C)	(MJ m^–2^ day^–1^)	(m^3^m^–3^)
A0	1.93 (0.71)	1.9	23.94	43.7	n.i.	0.16 (0.02)
A1	2.66 (0.98)	2.74	28.04	46.6	26.77	0.09 (0.03)
A2	1.35 (0.67)	−5.82	19.5	39.4	25.04	0.41 (0.02)
C1	1.01 (0.31)	−4.96	17.56	37.66	29.51	0.33 (0.05)
C2	1.40 (0.41)	−1.03	20.96	38.59	29.51	0.34 (0.06)
F1	1.16 (0.46)	1.5	22.86	37.95	19.56	0.40 (0.06)
G2	1.01 (0.41)	7.9	25.01	40.2	n.i.	0.45 (0.04)
I1	1.46 (0.54)	2.71	25.04	38.9	19.67	0.20 (0.07)
mean	1.498	0.62	22.86	40.37	25.01	0.29

*Mean VPD (SD in brackets) and minimum mean and maximum temperature of the air measured at the orchard canopy level; global radiation of the period (global radiation data from National Aeronautics and Space Administration Prediction of Worldwide Energy Resources (https://power.larc.nasa.gov/) and volumetric water content (VWC) of the soil measured with TDR soil probes.*

### Conductance of Orchard Systems

**FIGURE 2:**
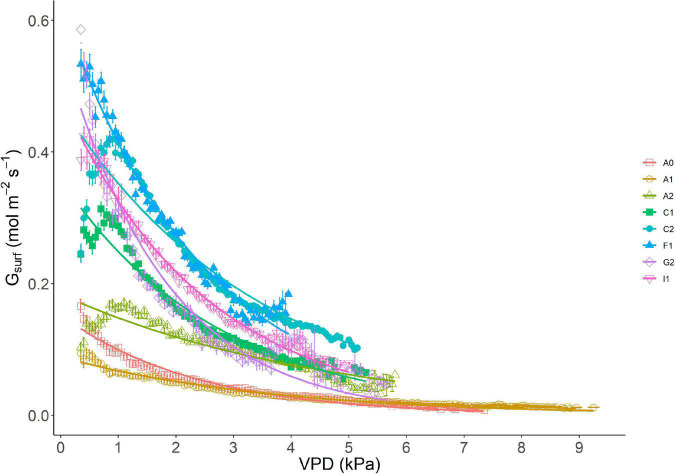
Response of *G*_*surf*_ to VPD in different sites (absolute values). Data are binned using 0.1 kPa bin size. SE per each interval of 0.1 kPa of VPD is shown on site-related markers.

The response curve of *G*_*surf*_ to VPD classes (bin size 0.1 kPa) showed an initial increase of *G*_*surf*_ at very low VPD, followed by the typical exponential decrease with higher VPD (Figure 2). Different maximum values of *G*_*surf*_ were recorded at site level and explain the behavior of different orchard systems with different LAI: *G*_*max*_ was very low in site A1 (*G*_*max*_ = 0.094 mol m^–2^s^–1^), but much higher in site G2 (*G*_*max*_ = 0.586 mol m^–2^s^–1^) (Table 3). The maximum canopy conductance *G*_*max*_ (Table 3) occurred on average at VPD = 0.57 kPa across sites, ranging between 0.35 and 1.1 kPa (Table 4). In Supplementary Figure 1, the same response curve is presented with *G*_*surf*_ normalized by leaf area. In this case, *G*_*max*_ differences collapse to a range of 0.1 mol m^–2^s^–1^, while in some sites (e.g., F1), the response to VPD per leaf area unit is highly reduced (Figure 1).

**TABLE 3:** Output of the linear model computed as lm = [Grel∼log(VPD) + log (VPD) × site, where *b* and *m* are the parameter of the equation *G*_*rel*_ = *m* × ln (VPD) + *b*.

*Site*	G*_*surf*_* = m* ln(VPD) + *b*						Gmax		G@1 kPa	
	*b*	*m*		*m* c.i.		*R* ^2^	(mol m^–2^s^–1^)		(mol m^–2^s^–1^)	
A0	0.095	−0.046	d	−0.047	−0.044	0.98	0.166	(0.377)	0.103	(0.05)
A1	0.067	−0.027	e	−0.027	−0.026	0.98	0.094	(0.082)	0.064	(0.039)
A2	0.124	−0.030	e	−0.036	−0.023	0.97	0.171	(0.081)	0.164	(0.089)
C1	0.266	−0.134	a	−0.138	−0.129	0.98	0.313	(0.151)	0.288	(0.087)
C2	0.404	−0.192	c	−0.197	−0.186	0.98	0.42	(0.14)	0.415	(0.121)
F1	0.397	−0.190	a	−0.197	−0.181	0.97	0.534	(0.516)	0.423	(0.186)
G2	0.305	−0.165	b	−0.174	−0.156	0.93	0.586	(0.41)	0.363	(0.303)
I1	0.318	−0.154	b	−0.157	−0.150	0.98	0.424	(0.208)	0.337	(0.125)

*Seasonal G_max_ and G_@1kPa_ are also reported per site with SD in brackets.*

*Significance level of m difference between sites (A0 as reference), CIs of the semi-logarithmic curve and adjusted-R squared at corresponding sites.*

**TABLE 4:** Mean values of VPD at *G*_*max*_ and at the lower and upper limit of the VPD_80_ interval, i.e., when *G*_*surf*_ = 80% of *G_*max*_.*

Site	VPD lower limit at 80% *G*_*surf*_	VPD at *G*_*max*_	VPD upper limit at 80% *G*_*surf*_	mean *G*_*surf*_ in interval VPD_80_
	(kPa)			(mol m^–^2s^–1^)
A0	0.15	0.35	0.55	0.15
A1	0.20	0.45	0.70	0.09
A2	0.45	1.10	1.75	0.15
C1	0.40	0.70	1.20	0.28
C2	0.50	0.90	1.45	0.39
F1	0.30	0.35	0.95	0.49
G2	0.20	0.35	0.50	0.52
I1	0.10	0.40	0.90	0.38
General mean	0.29	0.58	1.00	0.30

*The mean absolute value of G_surf_ within the interval of VPD_80_ is reported.*

The response of *G*_*surf*_ to VPD resulted well fitted in all the sites by a semi-log transformation of the type y = -*m* × log (x) + *b* as suggested in Oren et al. (1999). Globally, the regression between surface conductance (*G*_*surf*_) and log (VPD) resulted highly significant (*R*^2^ = 0.978) meaning that *G*_*surf*_ is strictly determined by the VPD measured at site level. The parameter *m*, i.e., the slope of the logarithmic function, was significantly different between sites (ANOVA, *p* < 0.01). Still, two main clusters were identified. The first included Australian sites A0, A1, and A2, where *m* values were between −0.027 and −0.046 describing a very low decrease of *G*_*surf*_ with VPD. The second cluster showed higher *m* values ranging from −0.134 in C1 to −0.192 in C2 (Table 3). Together with higher *G*_*max*_, these orchard systems have a higher GEC of the canopy together with a faster decrease of *G*_*rel*_ with an increase in VPD.

**FIGURE 3:**
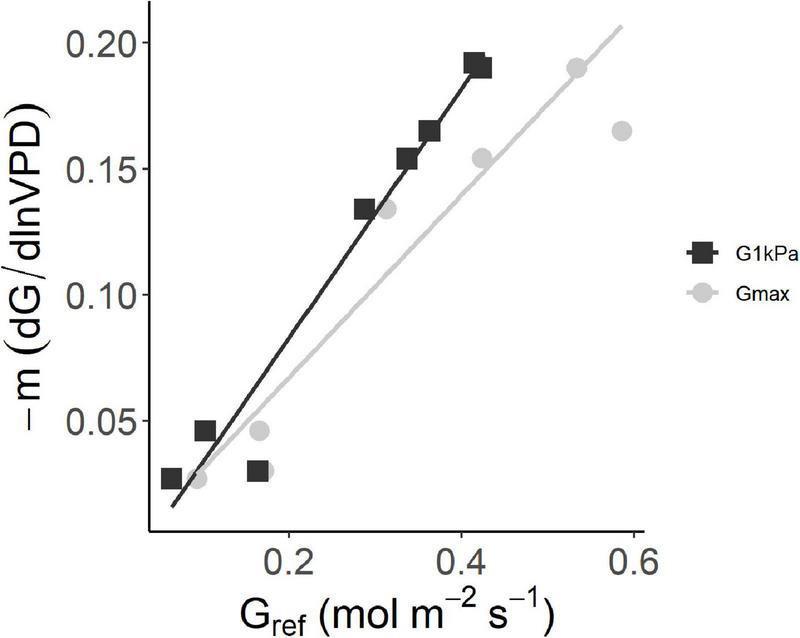
The sensitivity of average stomatal conductance at site level to increasing vapor pressure deficit (– dG/d lnVPD) as a function of the canopy stomatal conductance at VPD = 1 kPa and at its maximum value. The two axes represent the slope and intercept of the relationship G = – *m* ln(VPD) – *b*. G@1 kPa: y = 0.50X–0.016 (*R*^2^ = 0.96; *G*_*max*_: y = 0.36X–0.005 (*R*^2^ = 0.85).

The clustering of site sensitivity emerges clearly in the regression between –*m* and the maximum canopy conductance. We compared the variation of the sensitivity parameter *–m* to *G*_*max*_ and *G_@1k*Pa*_* (Figure 3). Even if a better correlation resulted from the linear regression of *–m* against *G_@1k*Pa*_* (*R*^2^ = 0.96, *p* < 0.001) with respect to *G*_*max*_ (*R*^2^ = 0.85, *p* < 0.01), the linear regressions are not significantly different from each other (*p* > 0.05). Thus, *G*_*max*_ can be used as reference value. Overall, lower sensitivity (lower *–m* values) corresponded to lower values of both the reference conductance *G*_*max*_ and *G_@1k*Pa*_*.

In addition, four sites with two different cultivars were selected to detect whether there was any clustering effect on the response of *G*_*surf*_ to VPD. Sites C2 and I1 were selected for cultivar TT and C1 and F1 for cultivar TG. However, we did not observe any difference related to the cultivar.

**FIGURE 4:**
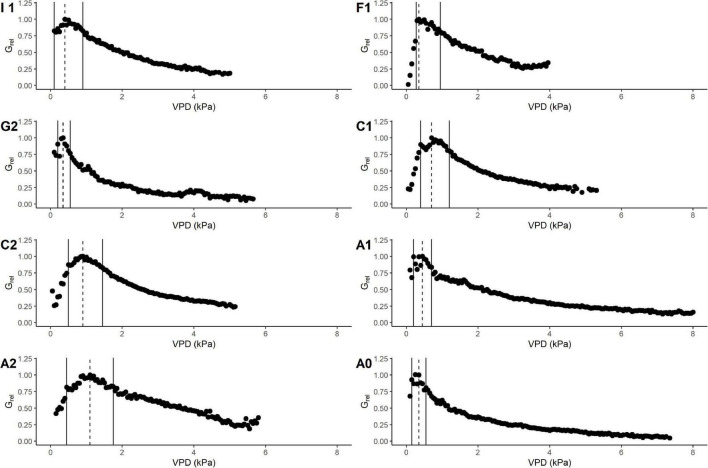
Response of *G*_*rel*_ to VPD class (kPa) in different sites. Study areas are marked with the corresponding site ID as described in “Materials and Methods” section. Markers refer to mean values of *G*_*rel*_ in each VPD class (0.1 kPa). Data are binned using 0.1 kPa bin size. Vertical dashed line shows the VPD value at maximum *G*_*rel*_, while the solid line shows the limits of VPD value at 80% of the *G*_*rel*_.

*G*_*max*_ was used to calculate the relative canopy conductance (*G*_*rel*_) in order to compare the pattern of conductance response to VPD between sites (Figure 4). The interval width of the VPD_80_, i.e., the interval of VPD in which *G*_*rel*_ is maintained ≥ 80% of its maximum was variable between sites. Even if the upper limit of VPD_80_ occurred on average at 1 kPa, there was high variability between sites. Sites A0, A1, and G2 resulted as having a narrower interval of VPD_80_ due to an initial pronounced peak in *G*_*rel*_ followed by a flatter decline; the upper limit of the VPD_80_ remained within 0.75 kPa in these sites (Table 4), where we registered the highest maximum and minimum air temperature. On the contrary, the rest of the sites showed a wider interval of VPD_80_ that reached the maximum value of 1.75 kPa in site A2, which is also the one with the lowest minimum temperature. These observations suggest that these plants might have acclimated to most extreme sites by changing their response to climate.

Finally, the mean *G*_*surf*_ value in each VPD_80_ resulted well correlated (*R*^2^ = 0.61) to interval with the fruit load per tree (maximum value over the 3 years) at site level (Supplementary Figure 2).

### Maximum Gas Exchange Capacity

**FIGURE 5:**
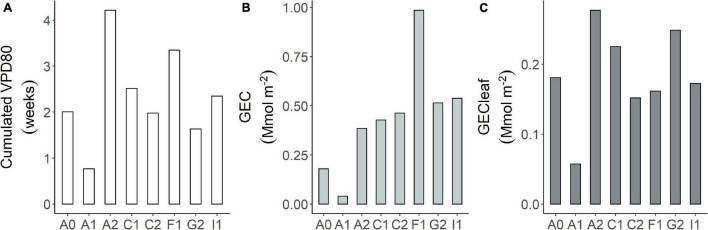
**(A)** Cumulated VPD_80_ expressed as weeks per site during the main growing season. As the growing season, we considered the period from May to August in the northern hemisphere and from November to February in the southern; maximum gas exchange capacity (GEC) per site: **(B)** GEC = n.VPD_80_ × *G*_*surf*_; **(C)** GEC_*leaf*_ = n.VPD_80_ × *G*_*surf*_ per unit of leaf area.

The sum of time with VPD_80_ calculated over the 4 central months of the growing season (May to August and November to February) amounted to about 4 weeks in site A2 and 3.5 in site F1 (Figure 5A). Site A1 appeared the less suitable site in terms of climatic conditions with less than 1 week per growing season in which *G*_*rel*_ is above 80% of its potential. Even if some sites have a higher frequency of VPD_80_ over the growing season, they might have low mean *G*_*surf*_ in this interval, as, for example, site A2 with 4 weeks of VPD_80_ but only *G_*surf*_* = 0.15 mol m^–2^s^–1^.

By combining these two characteristics per site, we obtained the maximum GEC of the orchard (Figure 5B). The GEC resulted the highest in site F1 because of the joint occurrence of high frequency of VPD_80_ and high mean *G*_*surf*_. Most of the other sites are similar in terms of GEC (about 0.45 Mmol m^–2^) because the differences in mean *G*_*surf*_ are compensated by the width of the VPD_80_ interval. Site A1 remains the site with the lowest GEC produced by the joint occurrence of very high VPD and very low mean *G*_*surf*_. These results represent the whole orchard response. Due to this, they implicitly include the effect of the orchard structure (i.e., density, LAI, etc.). If we consider the GEC per unit of leaf area (Figure 5C), the differences decrease and they are parallel to variations of the mean VPD80.

## Discussion

The comparative study of a species grown in different sites and under different orchard managements is a useful experimental setting to understand orchard responses under climate warming and VPD rise in the field. These study areas had different mean daily VPD during the growing season and despite irrigation, the soil water content was also affected. Australian sites A0 and A1 showed the most severe evaporative conditions with peaks of 5.2 kPa and daily mean temperature up to 46.6°C. These sites could represent the worst climatic scenario in which hazelnut can grow, together with a soil water content of 0.09 m^3^ m^–3^. Italy was also an area with high VPD together with the Australian site A2 located at a higher altitude with respect to A0 and A1. All these regions combined with high daily global radiation in the southern hemisphere represent a very challenging environment for hazelnut that potentially limits the canopy conductance and growth. At present, France (F1) and Chile (C1 and C2) are in a milder condition, but climatic projections forecast increasing atmospheric water demand at global level (i.e., higher VPD) with extreme cases in the southern hemisphere related to the dry phase of El Niño cycles (Zhang et al., 2015; Barkhordarian et al., 2019). Thus, even milder sites may become challenging places to grow hazelnut and it is crucial to know how orchards will respond to these changes in order to adapt the cultivation systems.

The hazelnut response of *G*_*surf*_ to VPD across sites showed some differences between orchards in terms of both the pattern and *G*_*max*_ values. Differences in *G*_*max*_ are representative of wide range of LAI, which developed in orchards; thanks to different management strategies (spacing and training system). Different orchard managements were reported to have a significant effect also on grapevines gas exchanges (Prieto et al., 2020). Because the *G*_*surf*_ at the leaf area level is similar between sites, orchards with higher LAI have higher total *G*_*max*_ at the community level. Indeed, orchards with lower LAI, as, for example, A0 and A1 (0.99 and 0.70, respectively), responded to the VPD similarly to grassland or savannah species (Grossiord et al., 2020). In other words, in sites A0 and A1, the whole tree stomata are not only more tightly closed at low VPD with respect to other sites, but also the velocity to close further is low per unit of increase in VPD. This effect is further enhanced by the low soil water content in site A1, where despite irrigation being homogeneously distributed, the higher percentage of sandy soil together with high temperatures severely compromised the water uptake capacity of trees. In these areas, the orchard management shall favor higher orchard LAI. Most of the other sites present values that are closer to deciduous forests. Indeed, absolute *G*_*max*_ agrees with findings on other broad leaves reported in the literature (Barradas et al., 2005; Tang et al., 2006; Bourne et al., 2015; Grossiord et al., 2020). Another factor that we find linked to the differences in *G*_*max*_ between sites is the fruit load. Orchards with higher fruit load had also higher *G*_*max*_. Accordingly, other studies on orange trees showed that low fruit loaded trees show < 40% stomatal conductance respect to fully loaded trees (Syvertsen et al., 2003). However, it has to be considered that fruit load and LAI, i.e., a proxy of the total photosynthetic capacity of the orchard, should be autocorrelated (Wünsche and Lakso, 2000).

The pattern of *G*_*surf*_ variation with VPD agrees with the theoretical framework presented by Oren et al. (1999) who described the response of *G*_*surf*_ to VPD by an exponential response curve, where the sensitivity is expressed by the parameter *–m* and higher *G*_*max*_ values predict higher sensitivity. Accordingly, Australian sites resulted in the less sensitive to increase in VPD and with very low *G_*max*_.*(average *m* = −0.03 mol m^–2^s^–1^ kPa^–1^), while the rest of the sites had an average *m* = −0.17 mol m^–2^s^–1^ kPa^–1^. This latter is close to what found for broadleaved forests (including hazelnut understory) according to Herbst et al. (2008) and for *Quercus alba* (Oren et al., 1999). Still, these two clusters of sites (Figure 3) highlight differences in the sensitivity within the same species, suggesting a moderate capacity to acclimate. In this sense, this study represents one of the few examples of conductance sensitivity measurements on the same species (Oren et al., 2001; Addington et al., 2004) and the first to compare a single species across continents.

The maximum canopy conductance *G*_*max*_ is an important parameter to define the optimal gas exchange capacity of a stand. *G*_*max*_ should be defined under favorable conditions for the species, i.e., non-limiting light, water availability, and optimum temperature. In this study, we are analyzing trees under standard irrigation and fertilization regimes, thus we can assume that *G*_*max*_ values are as close as possible to the potential for the specific site. Thus, *G*_*max*_ can be a reference value to determine the species sensitivity to the increasing VPD. Often, the reference G is *G*_*surf*_ at 1 kPa because it is difficult to obtain continuous and reliable measurements for low VPD. However, in our data collection, we could benefit from a long and continuous data series from sap flow measurements. In this study, *G*_*max*_ was observed to range widely across sites from A1 (0.091 mol m^–2^s^–1^) to G2 (0.58 mol m^–2^s^–1^), occurring on average at 0.57 kPa, thus at a lower VPD compared to the reference of 1 kPa proposed by Oren et al. (1999). Other authors such as Tang et al. (2006) and Herbst et al. (2008) observed similar values of *G*_*max*_ at VPD < 1 kPa in few temperate broadleaves including forests with hazelnut understory, while many fruit trees as olive, walnut, and apple reach *G*_*max*_ at higher VPD (1.5–2 kPa) (Rosati et al., 2006; Massonnet et al., 2007; Rodriguez-Dominguez et al., 2019). In this perspective, hazelnut can be considered as a species with a water-saving behavior, which typically takes advantage of low VPD to maximize stomata opening and the carbon uptake.

**FIGURE 6:**
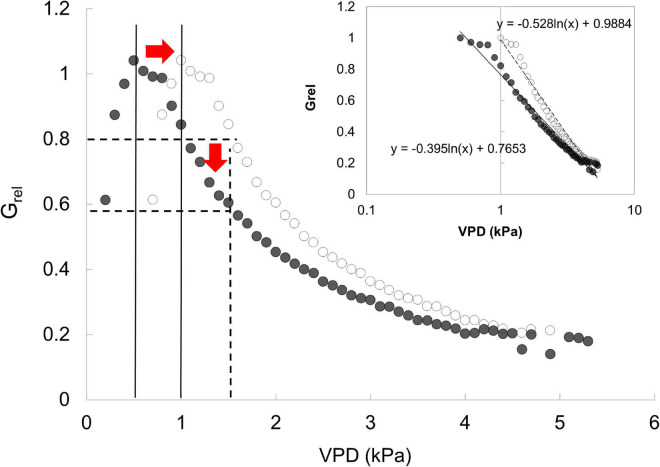
Simulation of the response of *G*_*rel*_ to VPD with *G*_*rel*_ calculated with two different maximum *G*. Inset presents the same data with x-axis log transformation. Black markers show the max *G*_*rel*_ at 0.5 kPa, the 0.8 × *G*_*rel*_ at 1 kPa and a slope of −0.39. White markers show max *G*_*rel*_ at 1 kPa, thus 0.8 × *G*_*rel*_ shifts toward the 1.5 kPa and the slope increases to about 0.13.

This behavior is also confirmed by the pattern of relative canopy conductance. In this study, not only the slope, but also the value of VPD at which *G*_*max*_ occurs is important to assess the sensitivity of a species in a specific stand structure. Thus, the sensitivity described in this study is not exactly comparable with that calculated with the reference *G_@1k*Pa*_* reported by Oren et al. (1999). Indeed, if *G*_*max*_ occurs at 0.5, we will obtain a less negative slope (i.e., lower sensitivity) respect to the case in which we consider the same *G*_*max*_ value at 1 kPa (Figure 6). We simulated a case in which *G*_*max*_ occurs at 1 kPa and we found *m* = −0.53, while when *G*_*max*_ occurs at 0.5, *m* = −0.39. This shows that force comparisons with *G_@1k*Pa*_* may show different results suggesting sensitivity higher than it really is. This might be the reason behind the lower slope value in the linear regression between the parameter *–m* and reference conductance with respect to found by Oren et al. (1999).

Indeed, the comparison of slopes between *G*_*surf*_ and d*G*_*surf*_/dln VPD expresses the actual degree of sensitivity of the same species between orchard management. Sites A1 and A0 had the lowest sensitivity (less negative slope). These sites are also those with more extreme events of VPD (up to 8–9 kPa). Thus, we can hypothesize that the lowest sensitivity is related to acclimation in these sites and this acclimation capacity is cultivar independent. Despite the cultivar is different in sites A0 and A1, the pattern of *G*_*surf*_ to VPD has a steep increase at low VPD followed by a reduction suggesting that trees might have acclimate their leaf characteristics (e.g., number of stomata, size of the stomata, or others) across the growing seasons and this would maximize their gas exchange.

The interval of VPD in which trees could maintain over 80% of their maximum tree conductance in most of the sites is below 1.7 kPa. In all the sites, values above 5 kPa reduce the conductance to the minimum. In A0 and A1, the interval is particularly narrow. This, together with the low sensitivity in these sites, is a further clue that suggests an acclimation capacity of trees. In other words, hazelnut might have responded to the high site VPD by reducing stomata opening and improve the water use efficiency. Also, G2 resulted in a site with narrow interval of VPD. This has in common with A0 and A1 very high minimum temperatures of the air in this study period even at low VPD. This might have somehow forced the plants to further reduce their maximum canopy conductance capacity at low VPD. The chance that hazelnut can develop acclimation to extreme climates is of great interest for orchard managers and for the food industry, which relies on world nut supplies. Indeed, if the mechanism of stomatal acclimation would miss, VPD variation due to climate change could reduce stomatal conductance by 10–50% (Ficklin and Novick, 2017).

When we consider the implication of the higher or lower sensitivity to the carbon economy of the plant, it is also important to consider the temporal scale (Martínez-Vilalta and Garcia-Forner, 2017). To test what is the impact of the VPD_80_ width on the total canopy conductance at the stand level, we estimated the total GEC of each orchard based on the occurrence of VPD_80_ during the growing season and the corresponding mean *G*_*surf*_ in this interval.

Most of the sites had on average 2 weeks (cumulated hours) per growing season in favorable climatic conditions for conductance maximization, i.e., VPD_80_ while for the rest of the season they are below the 80% of *G*_*max*_. F1 and A2 had more than 3 weeks suggesting that from the climatic perspective, these are very promising sites for hazelnut, while A1 has a very limited amount of time during the growing season to optimize the conductance. The situation of site A1 is further worsened by the extremely low *G_*surf*_* in the interval of VPD_80_, which makes the total GEC of the site undesirable. While A1 remains an extreme case, the compensation between the occurrences of VPD_80_ and *G_*surf*_* supports the hypothesis of some acclimation capacity of the species, which leads to a quite even GEC at site level (about 0.45 Mmol m^–2^). This acclimation might have been favored by the orchard management that favored a compensatory LAI through pruning and tree density. The extremely high GEC in site F1 is indeed related to the high LAI, close to a forest one, combined with very frequent VPD_80_. When we normalize GEC by LAI, we get the leaf area unit gas exchange, independently to the orchard structure. What is clear is that the GEC_leaf is highly related to the frequency of VPD_80_, but the orchard structures with higher LAI have, as a consequence, higher whole canopy gas exchange.

If the occurrence of VPD_80_ remains stable in future growing seasons, we can expect most sites to maintain a good level of potential productivity at comparable growing conditions. Still changes in VPD might overturn the suitability of some areas. In this perspective, VPD must be monitored. At the same time, the orchard management shall favor high *G*_*surf*_. This result can be achieved by increasing the LAI by favoring higher trees or by increasing the density where trees are small or still in the early productive stage. Indeed, the higher the fruit load, the higher the *G*_*surf*_ (Syvertsen et al., 2003). We believe that by collecting longer data series of fruit load combined with physiological and structural parameters of different orchards is a paramount goal to set in the future studies, perhaps toward modeling techniques as proposed by Prieto et al. (2020).

These results on suboptimal interval of VPD combined with the whole tree conductance (potential assimilation capacity) are the basis for a new global perspective on crop management. Indeed, they can be the basis for the construction of physiologically-based suitability maps: by using the projections of VPD conditions (Akpoti et al., 2019), it would be possible to roughly estimate the potential reduction of gas exchange and productivity in a given site.

Nonetheless, this study might provide guidelines for the management of existing orchards. We expect that the more the orchard is structured to maintain a range of favorable VPD conditions (within the VPD_80_ range), the higher the carbon stocked in trees available for nut production will be. Indeed, the canopy structure variation may largely influence the water use efficiency of orchards (Cohen and Fuchs, 1987; Cohen and Naor, 2002).

## Data Availability Statement

The raw data supporting the conclusions of this article will be made available by the authors, without undue reservation.

## Author Contributions

TA and GP discussed and developed the idea and underlying rationale. GP elaborated the data, proposed the analytical approach, and wrote the first draft. VC was in charge of the technical support of all the devices from the installation phase to the maintenance and dismantling, created the thermal dissipation probes (Lab of the University of Padova, Department of Territorio e Sistemi Agro-Forestali), downloaded, and prepared the raw data. All the authors commented and revised the final version of the manuscript.

## Conflict of Interest

This study received funding from *Hazelnut Company, Division of Ferrero Group.* The funder had the following involvement with this study: the study design and the location of study areas were agreed with the funder, besides the funder partly supported the field data collection. The authors declare that the research was conducted in the absence of any commercial or financial relationships that could be construed as a potential conflict of interest. The handling editor declared a past co-authorship with one of the authors ES.

## Publisher’s Note

All claims expressed in this article are solely those of the authors and do not necessarily represent those of their affiliated organizations, or those of the publisher, the editors and the reviewers. Any product that may be evaluated in this article, or claim that may be made by its manufacturer, is not guaranteed or endorsed by the publisher.

## References

[B1] Addington R. N. Mitchell R. J. Oren R. Donovan L. A. (2004). Stomatal sensitivity to vapor pressure deficit and its relationship to hydraulic conductance in *Pinus palustris*. *Tree Physiol.* 24 561–569. 10.1093/treephys/24.5.561 14996660

[B2] Akpoti K. Kabo-bah A. T. Zwart S. J. (2019). Agricultural land suitability analysis: state-of-the-art and outlooks for integration of climate change analysis. *Agric. Syst.* 173 172–208. 10.1016/j.agsy.2019.02.013

[B3] Arneth A. Kelliher F. M. Bauer G. Hollinger D. Y. Byers J. N. Hunt J. E. (1996). Environmental regulation of xylem sap flow and total conductance of *Larix gmelinii* trees in eastern Siberia. *Tree Physiol.* 16 247–255. 14871769 10.1093/treephys/16.1-2.247

[B4] Barkhordarian A. Saatchi S. S. Behrangi A. Loikith P. C. Mechoso C. R. (2019). A recent systematic increase in vapor pressure deficit over tropical South America. *Sci. Rep.* 9 1–12. 10.1038/s41598-019-51857-8 31653952 PMC6814800

[B5] Barradas V. L. Nicolás E. Torrecillas A. Alarcón J. J. (2005). Transpiration and canopy conductance in young apricot (Prunus armenica L.) trees subjected to different PAR levels and water stress. *Agric. Water Manag.* 77, 323–333.

[B6] Bourne A. E. Haigh A. M. Ellsworth D. S. (2015). Stomatal sensitivity to vapour pressure deficit relates to climate of origin in *Eucalyptus* species. *Tree Physiol.* 35 266–278. 10.1093/treephys/tpv014 25769338

[B7] Breshears D. D. Adams H. D. Eamus D. McDowell N. G. Law D. J. Will R. E. (2013). The critical amplifying role of increasing atmospheric moisture demand on tree mortality and associated regional die-off. *Front. Plant Sci.* 4:266. 10.3389/fpls.2013.00266 23935600 PMC3731633

[B8] Challinor A. J. Wheeler T. R. (2008). Crop yield reduction in the tropics under climate change: processes and uncertainties. *Agric. For. Meteorol.* 148 343–356. 10.1016/j.agrformet.2007.09.015

[B9] Chapin F. S. III Matson P. A. Mooney H. A. (2002). *Principles of Terrestrial Ecosystem Ecology.* New York, NY: Springer-Verlag, 298–300.

[B10] Cohen S. Fuchs M. (1987). The distribution of leaf area, radiation, photosynthesis and transpiration in a Shamouti orange hedgerow orchard. part I. leaf area and radiation. *Agric. For. Meteorol.* 40 123–144. 10.1016/0168-1923(87)90002-5

[B11] Cohen S. Naor A. (2002). The effect of three rootstocks on water use, canopy conductance and hydraulic parameters of apple trees and predicting canopy from hydraulic conductance. *Plant Cell Environ.* 25 17–28.

[B12] Eamus D. Boulain N. Cleverly J. Breshears D. D. (2013). Global change-type drought-induced tree mortality: vapor pressure deficit is more important than temperature per se in causing decline in tree health. *Ecol. Evol.* 3 2711–2729. 10.1002/ece3.664 24567834 PMC3930053

[B13] Fernández M. E. Gyenge J. Schlichter T. (2009). Water flux and canopy conductance of natural versus planted forests in Patagonia, South America. *Trees Struct. Funct.* 23, 415–427. 10.1007/s00468-008-0291-y

[B14] Ficklin D. L. Novick K. A. (2017). Historic and projected changes in vapor pressure deficit suggest a continental-scale drying of the United States atmosphere. *J. Geophys. Res.* 122 2061–2079. 10.1002/2016JD025855

[B15] Granier A. (1985). Une nouvelle methode pour la measure du flux de seve brute dans le tronc des arbres. *Ann. Des Sci. For.* 42 193–200. 10.1051/forest:19850204

[B16] Grossiord C. Buckley T. N. Cernusak L. A. Novick K. A. Poulter B. Siegwolf R. T. W. (2020). Plant responses to rising vapor pressure deficit. *New Phytol.* 226 1550–1566. 10.1111/nph.16485 32064613

[B17] Herbst M. Rosier P. T. W. Morecroft M. D. Gowlng D. J. (2008). Comparative measurements of transpiration and canopy conductance in two mixed deciduous woodlands differing in structure and species composition. *Tree Physiol.* 28 959–970. 10.1093/treephys/28.6.959 18381276

[B18] Hogg E. H. Hurdle P. A. (1997). Sap flow in trembling aspen: implications for stomatal responses to vapor pressure deficit. *Tree Physiol.* 17 501–509. 10.1093/treephys/17.8-9.501 14759823

[B19] Hsiao J. Swann A. L. S. Kim S. H. (2019). Maize yield under a changing climate: the hidden role of vapor pressure deficit. *Agric. For. Meteorol.* 279:107692. 10.1016/j.agrformet.2019.107692

[B20] IPCC (2018). “Summary for policymakers,” in *Global Warming of 1.5°C. An IPCC Special Report on the Impacts of Global Warming of 1.5°C Above Pre-industrial Levels and Related Global Greenhouse Gas Emission Pathways, in the Context of Strengthening the Global Response to the Threat of Climate Change, Sustainable Development, and Efforts to Eradicate Poverty*, eds Masson-Delmotte V. Zhai P. Pörtner H.-O. Roberts D. Skea J. Shukla P. R. (Geneva: World Meteorological Organization), 32.

[B21] Jarvis P. G. McNaughton K. G. (1986). Stomatal control of transpiration: scaling up from leaf to region. *Adv. Ecol. Res.* 15 1–49. 10.1016/S0065-2504(08)60119-1

[B22] Jones H. G. (1992). *Plants and Microclimate?: A Quantitative Approach to Environmental Plant Physiology.* Cambridge: Cambridge University Press.

[B23] Jones H. G. (1999). Use of thermography for quantitative studies of spatial and temporal variation of stomatal conductance over leaf surfaces. *Plant Cell Environ.* 22 1043–1055. 10.1046/j.1365-3040.1999.00468.x

[B24] Köstner B. Biron P. Siegwolf R. Granier A. (1996). Estimates of water vapor flux and canopy conductance of Scots pine at the tree level utilizing different xylem sap flow methods. *Theor. Appl. Climatol.* 53 105–113. 10.1007/BF00866415

[B25] Köstner B. M. Schulze E. D. Kelliher F. M. Hollinger D. Y. Byers J. N. Hunt J. E. (1992). Transpiration and canopy conductance in a pristine broad-leaved forest of *Nothofagus*: an analysis of xylem sap flow and eddy correlation measurements. *Oecologia* 91 350–359. 10.1007/BF00317623 28313542

[B26] Lobell D. B. Schlenker W. Costa-Roberts J. (2011). Climate trends and global crop production since 1980. *Science* 333 616–620. 10.1126/science.1204531 21551030

[B27] Martínez-Vilalta J. Garcia-Forner N. (2017). Water potential regulation, stomatal behaviour and hydraulic transport under drought: deconstructing the iso/anisohydric concept. *Plant Cell Environ.* 40 962–976. 10.1111/pce.12846 27739594

[B28] Massonnet C. Costes E. Rambal S. Dreyer E. Regnard J. L. (2007). Stomatal regulation of photosynthesis in apple leaves: evidence for different water-use strategies between two cultivars. *Ann. Bot.* 100 1347–1356. 17901058 10.1093/aob/mcm222 PMC2759240

[B29] McDowell N. G. Ryan M. G. Zeppel M. J. B. Tissue D. T. (2013). Feature: improving our knowledge of drought-induced forest mortality through experiments, observations, and modeling. *New Phytol.* 200 289–293. 10.1111/nph.12502 24050629

[B30] Medrano H. Tomás M. Martorell S. Flexas J. Hernández E. Rosselló J. (2015). From leaf to whole-plant water use efficiency (WUE) in complex canopies: limitations of leaf WUE as a selection target. *Crop J.* 3 220–228. 10.1016/j.cj.2015.04.002

[B31] Monteith J. L. Unsworth M. H. (2013). *Principles of Environmental Physics. Plants, Animals and the Atmosphere*, 4th Edn. Cambridge, MA: Academic Press.

[B32] Novick K. A. Ficklin D. L. Stoy P. C. Williams C. A. Bohrer G. Oishi A. C. (2016). The increasing importance of atmospheric demand for ecosystem water and carbon fluxes. *Nat. Clim. Chang.* 6 1023–1027. 10.1038/nclimate3114

[B33] Oren R. Phillips N. Katul G. Ewers B. E. Pataki D. E. (1998). Scaling xylem sap flux and soil water balance and calculating variance: a method for partitioning water flux in forests. *Ann. Sci. For.* 55 191–216. 10.1051/forest:19980112

[B34] Oren R. Sperry J. S. Ewers B. E. Pataki D. E. Phillips N. Megonigal J. P. (2001). Sensitivity of mean canopy stomatal conductance to vapor pressure deficit in a flooded *Taxodium distichum* L. forest: hydraulic and non-hydraulic effects. *Oecologia* 126 21–29. 10.1007/s004420000497 28547434

[B35] Oren R. Sperry J. S. Katul G. G. Pataki D. E. Ewers B. E. Phillips N. (1999). Survey and synthesis of intra- and interspecific variation in stomatal sensitivity to vapour pressure deficit. *Plant. Cell Environ.* 22 1515–1526. 10.1046/j.1365-3040.1999.00513.x

[B36] Pasqualotto G. Carraro V. Menardi R. Anfodillo T. (2019). Calibration of granier-type (TDP) sap flow probes by a high precision electronic potometer. *Sensors* 19:2419. 10.3390/s19102419 31137901 PMC6566514

[B37] Pisetta M. (2012). *Relazioni Idriche in Nocciolo (Corylus avellana L.).* Ph.D. thesis. Padova: University of Padova.

[B38] Prieto J. A. Louarn G. Perez Peña J. Ojeda H. Simonneau T. Lebon E. (2020). A functional-structural plant model that simulates whole- canopy gas exchange of grapevine plants (*Vitis vinifera* L.) under different training systems. *Ann. Bot.* 126 647–660. 31837221 10.1093/aob/mcz203 PMC7489073

[B39] Rodriguez-Dominguez C. M. Hernandez-Santana V. Buckley T. N. Fernández J. E. Diaz-Espejo A. (2019). Sensitivity of olive leaf turgor to air vapour pressure deficit correlates with diurnal maximum stomatal conductance. *Agric. For. Meteorol.* 27 156–165.

[B40] Romano B. Mandrioli P. Puppi G. Zanotti A. Botarelli L. Sacchetti V. (1998). *Guida al Rilevamento dei Giardini Fenologici Italiani. Progetto Finalizzato Phenagri: Fenologia per L’agricoltura; Sottoprogetto 2: Fenologia Delle Piante Arboree.* Roma.

[B41] Rosati A. Metcalf S. Buchner R. Fulton A. Lampinen B. (2015). Tree water status and gas exchange in walnut under drought, high temperature and vapour pressure deficit. *J. Hortic. Sci. Biotechnol.* 81 415–420.

[B42] Roversi A. (2014). How many mineral nutrients does a hazelnut orchard take up annually from the soil? *Acta Hortic.* 1052, 201–206.

[B43] Sanginés de Cárcer P. Vitasse Y. Peñuelas J. Jassey V. E. J. Buttler A. Signarbieux C. (2018). Vapor–pressure deficit and extreme climatic variables limit tree growth. *Glob. Chang. Biol.* 24 1108–1122. 10.1111/gcb.13973 29105230

[B44] Schulze E. D. Lange O. L. Buschbom U. Kappen L. Evenari M. (1972). Stomatal responses to changes in humidity in plants growing in the desert. *Planta* 108 259–270. 10.1007/BF00384113 24473858

[B45] Syvertsen J. P. Goñi C. Otero A. (2003). Fruit load and canopy shading affect leaf characteristics and net gas exchange of ‘spring’ navel orange trees. *Tree Physiol.* 23 899–906. 14532013 10.1093/treephys/23.13.899

[B46] Tang J. Bolstad P. V. Ewers B. E. Desai A. R. Davis K. J. Carey E. V. (2006). Sap flux-upscaled canopy transpiration, stomatal conductance, and water use efficiency in an old growth forest in the Great Lakes region of the United States. *J. Geophys. Res. Biogeosci.* 111 1–12. 10.1029/2005JG000083

[B47] Will R. E. Wilson S. M. Zou C. B. Hennessey T. C. (2013). Increased vapor pressure deficit due to higher temperature leads to greater transpiration and faster mortality during drought for tree seedlings common to the forest-grassland ecotone. *New Phytol.* 200 366–374. 10.1111/nph.12321 23718199

[B48] Wünsche J. N. Lakso A. N. (2000). The relationship between leaf area and light interception by spur and extension shoot leaves and apple orchard productivity. *Hortscience* 35 1202–1206.

[B49] Yuan W. Zheng Y. Piao S. Ciais P. Lombardozzi D. Wang Y. (2019). Increased atmospheric vapor pressure deficit reduces global vegetation growth. *Sci. Adv.* 5:1396. 10.1126/sciadv.aax1396 31453338 PMC6693914

[B50] Zhao C. Liu B. Piao S. Wang X. Lobell D. B. Huang Y. (2017). Temperature increase reduces global yields of major crops in four independent estimates. *Proc. Natl. Acad. Sci. U.S.A.* 114 9326–9331. 10.1073/pnas.1701762114 28811375 PMC5584412

[B51] Zhang K. Kimball J. S. Nemani R. R. Running S. W. Hong Y. Gourley J. J. (2015). Vegetation greening and climate change promote multidecadal rises of global land evapotranspiration. Sci. Rep. 5:15956. 10.1038/srep15956 26514110 PMC4626800

