# Supplementary material for: Assessment of Canopy Conductance Responses to Vapor Pressure Deficit in Eight Hazelnut Orchards Across Continents
Source: Front Plant Sci. 2021 Dec 8;12:767916. doi: 10.3389/fpls.2021.767916 (PMC8692988; doi:10.3389/fpls.2021.767916)
Supplement: Supplementary file 1 [file Data_Sheet_1.PDF]

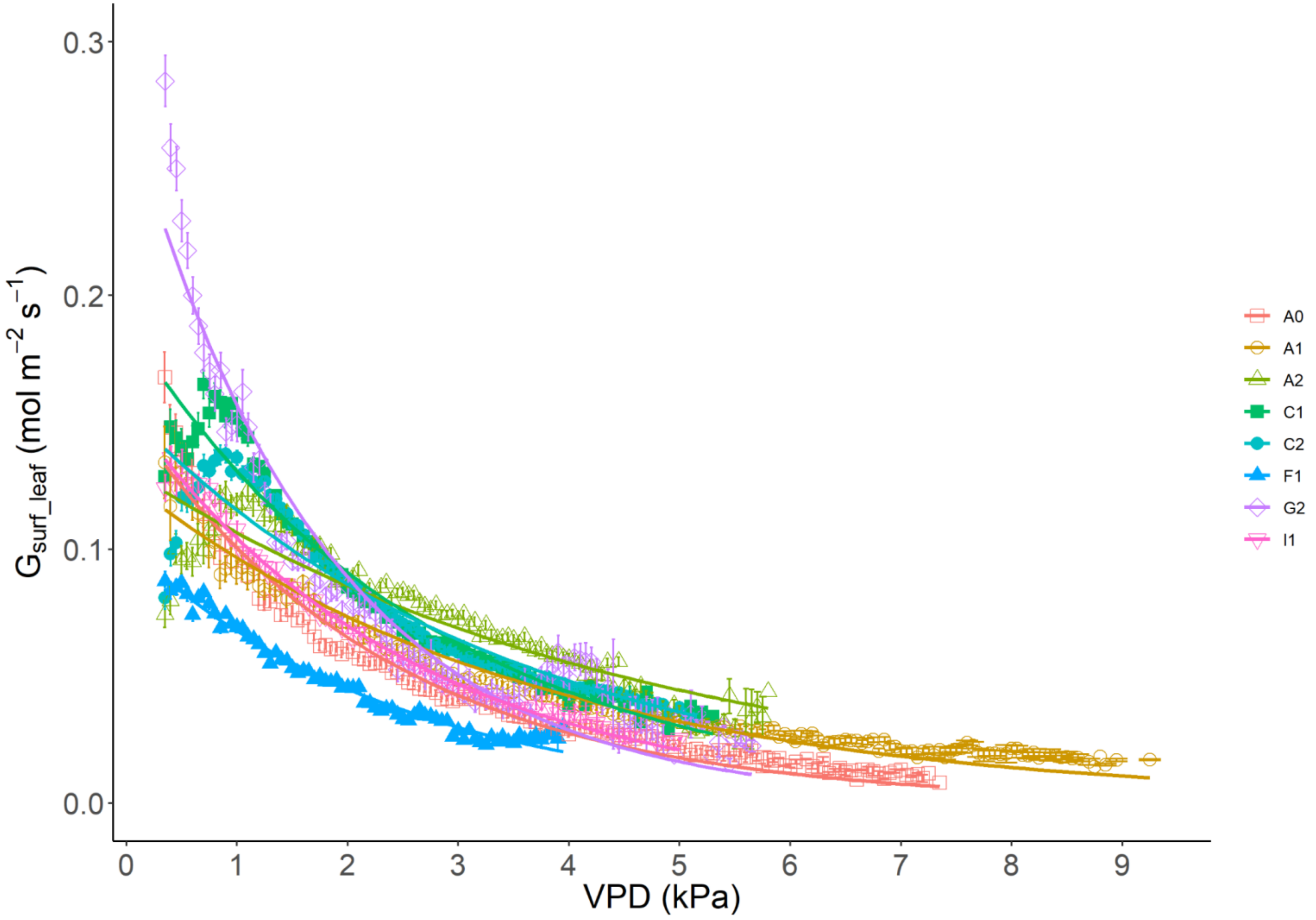

Supplementary Figure 1. Response of G_{surf} per unit of leaf area to VPD in different sites (absolute values). Data are binned using 0.1 kPa bin size. SE per each interval of 0.1 kPa of VPD is shown on site-related markers
