# Supplementary material for: Assessment of Canopy Conductance Responses to Vapor Pressure Deficit in Eight Hazelnut Orchards Across Continents
Source: Front Plant Sci. 2021 Dec 8;12:767916. doi: 10.3389/fpls.2021.767916 (PMC8692988; doi:10.3389/fpls.2021.767916)
Supplement: Supplementary file 2 [file Data_Sheet_2.PDF]

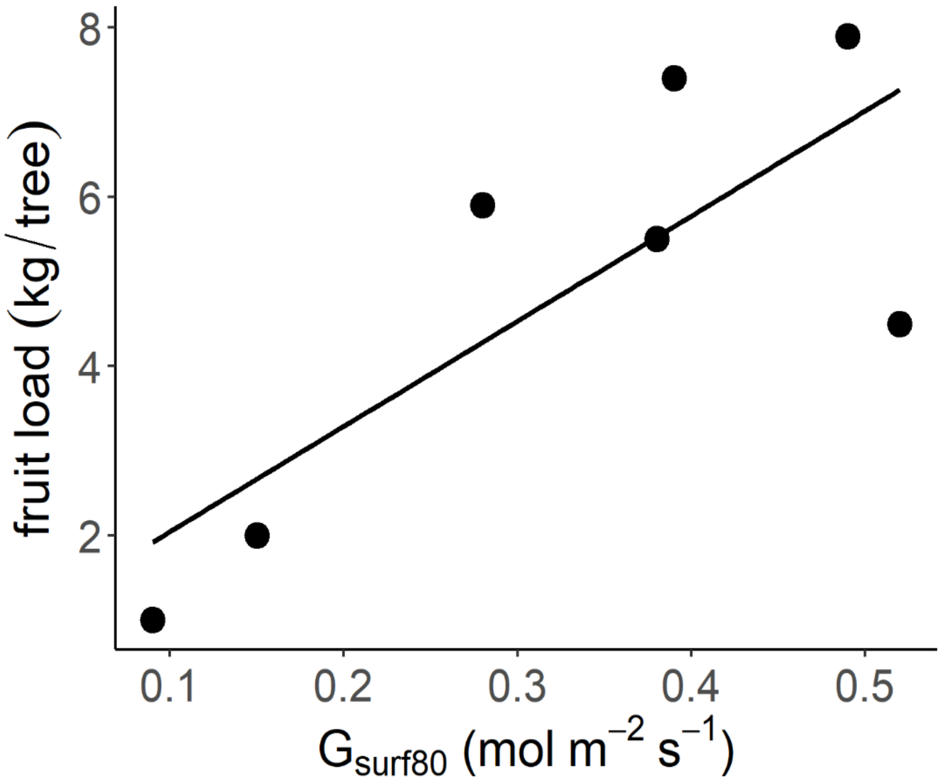

Supplementary Figure 2. Correlation between mean G_{surf} in the interval of VPD 80 and the mean fruit load per site (avaerage values). The linear correlation is expressed by the equation y = 12.427x + 0.8026, R^2 = 0.6129
